# [Retracted] Lidocaine inhibits the progression of retinoblastoma *in vitro* and *in vivo* by modulating the miR-520a-3p/EGFR axis

**DOI:** 10.3892/mmr.2025.13669

**Published:** 2025-09-01

**Authors:** Weiyi Xia, Libo Wang, Dongyi Yu, Xing Mu, Xin Zhou

Mol Med Rep 20: 1333–1342, 2019; DOI: 10.3892/mmr.2019.10363

Following the publication of this paper, it was drawn to the Editor's attention by a concerned reader that flow cytometric (FCM) assay data featured in Figs. 2 and 7B were strikingly similar to FCM data which were ultimately published in a number of other papers in different journals that were written by different authors at different research institutes, including a paper that was submitted on an earlier date to the journal *Experimental and Therapeutic Medicine.*

Owing to the fact that the contentious data in the above article were found to be strikingly similar to data that have appeared elsewhere in other papers in the scientific literature, the Editor of *Molecular Medicine Reports* has decided that this paper should be retracted from the Journal. After contacting the authors, they accepted the decision to retract the paper. The Editor apologizes to the readership for any inconvenience caused.

